# Peroxynitrite decomposition catalyst ameliorates renal damage and protein nitration in cisplatin-induced nephrotoxicity in rats

**DOI:** 10.1186/1471-2210-4-20

**Published:** 2004-09-30

**Authors:** Yolanda I Chirino, Rogelio Hernández-Pando, José Pedraza-Chaverrí

**Affiliations:** 1 Departamento de Biología, Facultad de Química, Edificio B, Segundo Piso, Lab 209, Ciudad Universitaria, UNAM, México D.F. México; 2 Departamento de Patología, Instituto Nacional de Ciencias Médicas y Nutrición "Salvador Zubirán" 14000, México, D.F. México

## Abstract

**Background:**

Oxidative stress is involved in cisplatin-nephrotoxicity. However, it has not completely established if reactive nitrogen species and nitrosative stress are involved in this experimental model. The purpose of this work was to study the role of peroxynitrite, a reactive nitrogen specie, in cisplatin-nephrotoxicity using the compound 5,10,15,20-tetrakis (4-sulfonatophenyl) porphyrinato iron (III) (FeTPPS), a soluble complex able to metabolize peroxynitrite.

**Results:**

In rats treated with cisplatin (a single intraperitoneal dose of 7.5 mg/kg body weight), renal nitrosative stress was made evident by the increase in 3-nitrotyrosine on day 3. In addition, cisplatin-induced nephrotoxicity was evident by the histological damage of proximal tubular cells and by the increase in (a) serum creatinine, (b) blood urea nitrogen, and (c) urinary excretion of N-acetyl-β-D-glucosaminidase and total protein. Cisplatin-induced nitrosative stress and nephrotoxicity were attenuated by FeTPPS-treatment (15 mg/kg body weight, intraperitoneally, every 12 hours for 3 days).

**Conclusions:**

Nitrosative stress is involved in cisplatin-induced nephrotoxicity in rats. Our data suggest that peroxynitrite is involved, at least in part, in cisplatin-induced nephrotoxicity and protein nitration.

## Background

Cisplatin (cis-dichlorodiammine-platinum II) is an effective antineoplastic agent in the treatment of various solid tumours [1] including cancers of the ovary, testis, bladder, head, neck, lung, cervix, and endometrium [2]. Nevertheless, its full clinical utility is limited due to some adverse side effects including acute renal failure. The major site of renal injury is the S3 segment of the proximal tubule, located in the outer stripe of the outer medulla of the kidney [1]. The production of reactive oxygen species (ROS) and oxidative stress in kidney have been implicated in the pathogenesis of cisplatin-induced renal injury [3]. It has been shown that superoxide anion (O_2_^•-^) [4], hydrogen peroxide (H_2_O_2_) [5], and hydroxyl radical (^•^OH) [6] are involved in cisplatin-induced nephrotoxicity. In addition, it has been found that renal lipid peroxidation [5,7] is increased and glutathione is decreased [8] in this experimental model. The involvement of oxidative stress is further supported by the fact that the antioxidants melatonin [9] and vitamins C and E [5,10] prevent cisplatin-induced nephrotoxicity. Interestingly, overexpression of heme oxygenase-1 ameliorates [11] and heme oxygenase-1 deficiency [12] aggravates renal damage induced by cisplatin, supporting additionally the involvement of oxidant stress in this experimental model.

On the other hand, the role of reactive nitrogen species (RNS) and nitrosative stress has been less explored in cisplatin-induced nephrotoxicity. In this context, it has been studied the role of nitric oxide (^•^NO) and nitric oxide synthase (NOS) [13-19]. It has been found that the renal content of total nitrate/nitrite is increased in cisplatin-treated rats [18,19] suggesting that ^•^NO production is enhanced in these animals. Furthermore, the inhibition of NOS by L-NAME [14] or by aminoguanidine [13] decreased renal damage induced by cisplatin, suggesting that ^•^NO is playing a toxic role in this experimental model. However, it is unknown if peroxynitrite (ONOO^-^), a RNS that is generated by the reaction of ^•^NO and O_2_^•-^, is involved in the renal damage induced by cisplatin. It has been shown that ONOO^-^, which is not a free radical, is involved in the pathogenesis of many diseases [20-25]. ONOO^- ^can react with different biomolecules including amino acids such as cysteine, methionine, tryptophan, and tyrosine leading to changes in protein structure and function [26]. ONOO^- ^has been shown to cause lipid peroxidation, chemical cleavage of DNA, and reduction in cellular defenses by oxidation of thiol pools [27].

In this work, we studied if ONOO^- ^is involved in the nephrotoxicity induced by cisplatin by using 5,10,15,20-tetrakis (4-sulfonatophenyl) porphyrinato iron (III) (FeTPPS). This compound is a water-soluble Fe (III) porphyrin complex that catalyzes rapid isomerization of ONOO^- ^to nitrate (NO_3_^-^) under physiologically relevant conditions (pH 7.4, 37°C) [28]. The cytoprotective actions of FeTPPS have been characterized [29].

## Results

### Body weight and urinary volume

Body weight decreased 8.5% in cisplatin (Cis) group on day 3 and FeTPPS tended to prevent this decrease in Cis+FeTPPS group, however there was no significative difference between Cis and Cis+FeTPPS groups. Body weight was similar in control (Ct), FeTPPS, and Cis+FeTPPS groups. Urinary volume was not significative difference among the four groups along the study and on day of sacrifice (Table 1).

**Table 1:** Body weight and urinary volume in the 4 groups of rats studied on day 3.

	Ct	Cis	FeTPPS	Cis+ FeTPPS
Body weight (g)	235 ± 5^a^	215 ± 4^b^	238 ± 3^a^	231 ± 4^a^
Urinary volume (mL/24 h)	5.7 ± 1.4^a^	7.4 ± 0.8^a^	3.5 ± 1.1^a^	7.4 ± 1.6^a^

Values are mean ± SEM. n = 6. Groups with different letter are significantly different (P < 0.05).

### Markers of glomerular and tubular damage

**Figure 1:**
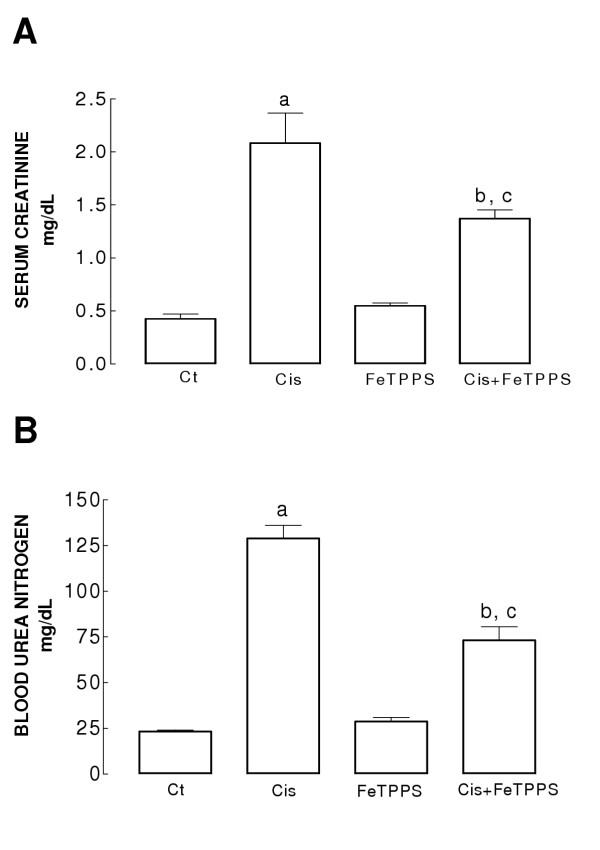
(A) Serum creatinine and (B) BUN on day 3 in the four groups of rats studied. Ct: control group, Cis: cisplatin group; FeTPPS: 5,10,15,20-tetrakis(4-sulfonatophenyl) porphyrinato iron (III) group, and Cis+FeTPPS: cisplatin+5,10,15,20-tetrakis(4'-sulfonatophenyl) porphyrinato iron (III) group. Data are mean ± SEM. n = 6. ^a^P < 0.001 vs. Ct; ^b^P < 0.001 vs. Ct, ^c^P < 0.05 vs Cis (Panel A); ^a^P < 0.001 vs. Ct; ^b^P < 0.001 vs. Ct, ^c^P < 0.001 vs Cis (Panel B). Serum creatinine and BUN increased in cisplatin group and FeTPPS prevented these increases in the Cis+FeTPPS group.

**Figure 2:**
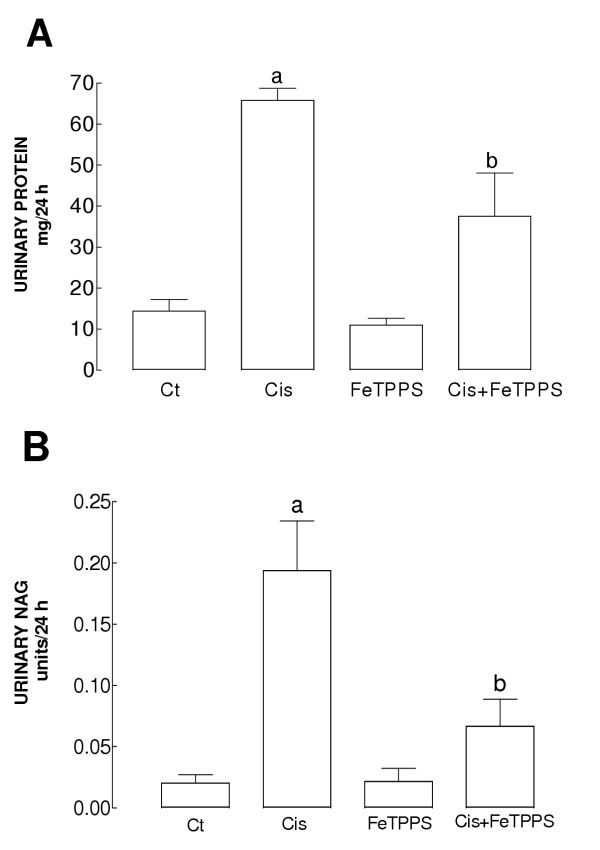
Urinary excretion of (A) total protein and (B) NAG on day 3 in the four groups of rats studied. Data are mean ± SEM. n = 5–6. ^a^P < 0.001 vs. Ct, ^b^P < 0.05 vs. Cis. Cisplatin-treated rats increased urinary excretion of total protein and NAG and these increases were prevented by FeTPPS administration in Cis+FeTPPS group.

Serum creatinine increased 4.9 times and blood urea nitrogen (BUN) increased 5.5 times in Cis group compared to control one (Fig 1). FeTPPS prevented partially the increase in serum creatinine and BUN levels in Cis+FeTPPS group. Cisplatin increased urinary excretion of total protein (4.6 times) and N-acetyl-β-D-glucosaminidase (NAG) (9.6 times) (Fig 2A and 2B). The increase in both parameters was prevented by FeTPPS in Cis+FeTPPS group (Fig 2). Serum creatinine, BUN, and urinary excretion of total protein and NAG were similar in Ct and FeTPPS groups (Figs 1 and 2).

### Histological analysis

**Figure 3:**
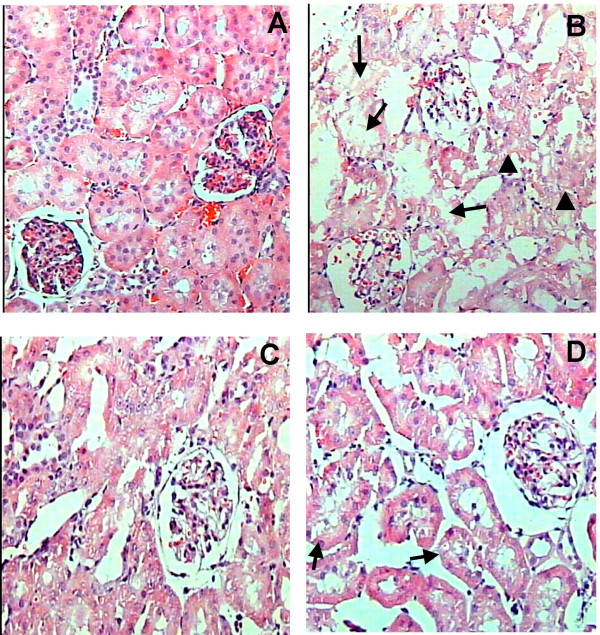
Representative histological abnormalities in the external cortical kidney area after three days of cisplatin administration and their partial prevention by FeTPPS. (A) Normal kidney histology from control rat. (B) After three days of cisplatin administration, many cortical convoluted tubules are revisted by necrotic epithelial cells (arrows) or vacuolated swell cells (arrow heads), glomeruli do not show apparent damage. (C) FeTPPS administration does not produce histological kidney abnormalities. (D) The administration of FeTPPS partially prevents the cytotoxic damage induced by cisplatin; arrows indicate middle cellular vacuolization of cortical convoluted tubules.

**Figure 4:**
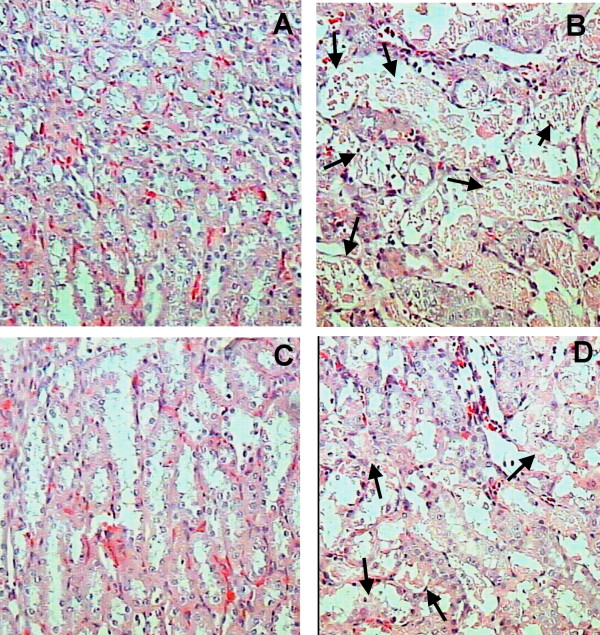
Representative histological abnormalities in the inner part of the cortical kidney after three days of cisplatin administration and their partial prevention by FeTPPS. (A) Normal kidney histology from control rat. (B) After three days of cisplatin administration, the straight portion of many cortical tubules are revisted by necrotic cells (arrows). (C) FeTPPS administration does not produce histological abnormalities. (D) The administration of FeTPPS partially prevents the cytotoxic damage induced by cisplatin; arrows indicate tubules with focal necrotic cells.

After three days of cisplatin-treatment, the epithelium from proximal convoluted tubules (tubules with small lumen area and taller epithelial cells) showed cytoplasmic vacuolization, intracellular edema and extensive damage which affected 87 ± 4% of their surface area (Fig 3B). The cisplatin toxic activity was higher in the straight portion of proximal convoluted tubules located in the inner area of the kidney cortex, where more than 90% of the epithelial surface suffered damage (Fig 4B). Interestingly, FeTPPS administration partially decreased the damaged area from 87 ± 4 to 44 ± 6% (p < 0.0001) in proximal convoluted tubules (Fig 3D) and from 93 ± 2 to 68 ± 10 (p < 0.0001) in the straight portion (Fig 4D). The administration of FeTPPS did not produce any histological alteration in the kidneys (Figs 3C and 4C). At the light microscopy level, glomeruli structure remained unchanged in all groups.

### Immunohistochemical localization of 3-nitro-L-tyrosine (3-NT)

**Figure 5:**
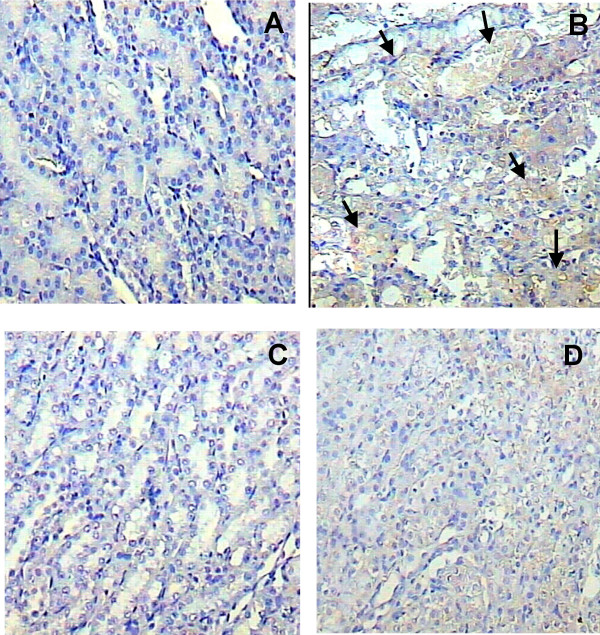
Nitrotyrosine (3-NT) expression determined by immunohistochemistry in the inner part of the cortical kidney after three days of cisplatin administration and its partial prevention by FeTPPS. (A) There is no 3-NT immunostaining in the kidney of control rat. (B) In contrast, three days after cisplatin administration there is a strong 3-NT expression in the necrotic cells from the straight portion of the proximal convoluted tubules (arrows). (C) FeTPPS administration does not induce 3-NT expression. (D) The administration of FeTPPS strongly decreases 3-NT expression induced by cisplatin-treatment (Cis+FeTPPS group).

A strong 3-NT immunostaining was observed in the straight portion of the proximal convoluted tubules located in the inner area of the kidney cortex of cisplatin-treated rats (Fig 5B). Interestingly, in the Cis+FeTPPS group, FeTPPS administration partially prevented the cisplatin toxic damage in the epithelium from the proximal convoluted tubules and its straight portion respectively, in coexistence with an evident decrease of 3-NT immunostaining (Fig 5D).

## Discussion

Cisplatin in an effective chemotherapeutic agent for a wide variety of tumors, nevertheless, nephrotoxicity is the major complication of this antineoplasic treatment [1]. The mechanism by which cisplatin causes renal damage is unclear, however, it has been postulated that oxidative stress is involved in this process [2,3,13,30]. The protective effect of overexpression of Mn-SOD [4] or the *in vivo *administration of some antioxidants such as vitamins C and E [5,10], melatonin [9], or selenium [31] in cisplatin-induced nephrotoxicity as well as the protective effect of tiron (a cell permeable O_2_^•- ^scavenger), pyruvate and catalase (H_2_O_2 _scavengers), and dimethylthiourea and thiourea (^•^OH scavengers) in renal proximal tubular epithelial cells (LLC-PK1 cells) treated with cisplatin also strongly support the role of ROS in cisplatin renal toxicity [30].

In contrast the role of ^•^NO and RNS in cisplatin-induced nephrotoxicity has not been completely established. It has been shown that the renal content of nitrate/nitrite is increased in cisplatin-treated rats suggesting that ^•^NO is increased in these animals [18,19]. In fact it has been shown that renal NOS activity is increased in cisplatin-treated rats [14]. In addition, the following two experiments suggest a toxic role of ^•^NO on cisplatin-induced renal toxicity: (a) aminoguanidine, an inhibitor of inducible NOS, decreased nephrotoxicity and prevented kidney lipid peroxidation and reduction of antioxidant enzymes induced by cisplatin [13], and (b) the administration of N(G)-nitro-L-arginine methyl ester (L-NAME), an inhibitor of NOS, reduced renal and gastrointestinal toxicity along with a significant inhibition in lipid peroxidation induced by cisplatin [14]. In contrast, Mansour *et al*. [15] and Li *et al*. [16] have found that L-NAME administration resulted in no protection and Saad *et al*. [17] found that this NOS inhibitor aggravates cisplatin-induced renal dysfunction. These data may suggest that ^•^NO is not playing a toxic role in cisplatin-induced nephrotoxicity. The above mentioned disagreement justify the performance of additional experiments to clarify the role of NOS and ^•^NO in cisplatin-induced nephrotoxicity.

On the other hand, ^•^NO is able to react with O_2 _^•- ^to produce ONOO^-^, which is a powerful oxidant more, reactive than its precursors, and has been implicated in an increasing list of diseases: hyperlipidemia [32], Alzheimer [20], acute renal ischemia [25], neurotoxicity induced by methamphetamine [33], and diabetes [23]. The ONOO^- ^decomposition catalyst FeTPPS is a water-soluble Fe (III) porphyrin complex able to block ONOO^- ^toxicity [28,29] and to protect against toxic insults in several experimental models. In focal cerebral ischemia-reperfusion in rats, massive production of ^•^NO and O_2_^•- ^results in continuous formation of ONOO^- ^even several hours after ischemia-reperfusion insult [34]. Significant reduction of 3-NT in brain sections and prominent neuroprotection was observed by FeTPPS (30 mg/kg) [34]. In a model of sepsis induced by injection of endotoxin (10 mg/kg) in rats, FeTPPS prevented the accumulation of ONOO^- ^as measured by plasma rhodamine fluorescence and heart 3-NT staining [35]. Interestingly, FeTPPS improved endotoxin-induced myocardial contractile dysfunction, which was associated with reduced degradation of nuclear factor kappa B inhibitory protein I-kappa-B, plasma TNF-alpha levels, and microvascular endothelial cell-leukocyte activation [35].

In this work it was found that FeTPPS partially prevented the increase in BUN and serum creatinine (markers of glomerular damage) and urinary excretion of NAG and total protein (markers of tubular damage) induced by cisplatin-treatment. The increase in urinary NAG and total protein excretion could be associated with necrosis of the proximal convoluted tubules, the primary site of drug accumulation [1]. FeTPPS prevented these alterations induced by cisplatin. This may be secondary to the ability of FeTPPS to catalyze the decomposition of ONOO^- ^which could be responsible, at least in part, of the alterations induced by cisplatin. This ameliorative effect of FeTPPS was associated with the decrease in 3-NT staining suggesting that ONOO^- ^is involved in protein nitration in cisplatin-nephrotoxicity. It is known that another RNS such as N_2_O_4_, HONOO, ^•^NO_2 _[36], and nitryl chloride (NO_2_Cl) [37], are involved in protein nitration. Nitryl chloride is formed by the reaction of NO_2_^- ^and HOCl-derived myeloperoxidase [37].

Studies in animals have established that tubular injury plays a central role in the reduction of glomerular filtration rate in acute tubular necrosis. Two major tubular abnormalities could be involved in the decrease in glomerular fucntion in cisplatin-treated rats: obstruction and backleak of glomerular filtrate. The alteration in glomerular function can not be attributed to structural damage since glomeruli structure is normal in cisplatin-treated rats. The alterations in glomerular function in cisplatin-treated rats may also be secondary to ROS [38] which induce mesangial cells contraction, altering the filtration surface area and modifying the ultrafiltration coefficient, factors that decrease the glomerular filtration rate. In addition our data suggest that ONOO^- ^may also be involved in the glomerular alterations in cisplatin-treated rats.

The increase in renal ONOO^- ^induced by cisplatin may be secondary to the increase in ^•^NO and O_2_^•- ^production. In fact, there are evidences of the renal increase in ^•^NO production in cisplatin nephrotoxicity [18,19] and O_2_^•- ^generation in cisplatin-treated LLC-PK1 cells [30]. The O_2_^•- ^increase in cisplatin-nephrotoxicity may be simply consequence of the mitochondrial dysfunction [39] and the decrease in superoxide dismutase activity [5].

## Conclusions

Nitrosative stress is involved in cisplatin-induced nephrotoxicity in rats. The ameliorative effect of FeTPPS on cisplatin-induced nephrotoxicity in rats was associated with the decrease in protein nitration suggesting that ONOO^- ^is involved in both protein nitration and nephrotoxicity in these animals.

## Methods

### Reagents

Cisplatin (catalogue # P-4394) was from Sigma-Aldrich (St. Louis MO, USA). FeTPPS (catalogue # 341492) was from (Calbiochem, San Diego, CA, USA). Rabbit anti-3-NT polyclonal antibodies (Catalogue # 06–284) were from Upstate (Lake Placid, NY, USA). Anti-rabbit Ig horseradish peroxidase antibodies (Catalogue # SAB-300) were purchased from Stressgen (Victoria BC, Canada). Commercial kits to measure creatinine and urea were from Spinreact (Girona, Spain). All other chemicals were reagent grade and commercially available.

### Experimental design

Male Wistar rats (Harlan Teklad, Mexico City, Mexico) initially weighing 200–250 g were used. Experimental work was approved by DGAPA (IN227103) and followed the guidelines of Norma Oficial Mexicana (NOM-ECOL-087-1995). All animals had free access to water and commercial rodent diet (Harlan Teklad, catalogue 2018S), and were randomly divided in four groups (n = 6 rats/group) as follows: (1) CT, injected intraperitoneally (i.p.) with isotonic saline solution; (2) Cis, treated with a single dose of cisplatin (7.5 mg/Kg b.w./i.p.) [40]; (3) FeTPPS, treated with FeTPPS (15 mg/kg/i.p./12 h) [32] for 3 days; and (4) Cis+FeTPPS, treated with Cis and with FeTPPS. During the study rats were maintained with a 12-h light:dark cycle in stainless steel metabolic cages to collect urine. On day 3, the animals were sacrificed by decapitation and blood was collected to obtain serum and to measure creatinine and BUN. Total protein and NAG were measured in 24-h urine. The kidneys were removed to obtain cortex samples for histological and immunohistochemical studies.

### Markers of glomerular and tubular damage

The markers of glomerular damage, creatinine and urea, were measured using commercial kits. BUN was obtained by correcting the urea value by a 2.14 factor [41]. As markers of tubular damage, we measured urinary excretion of NAG and total protein. NAG activity was measured using p-nitrophenyl-N-acetyl-β-D-glucosaminide as substrate and total protein was measured by a turbidimetric method [42].

### Histological analysis

Thin slices of kidney tissue with cortex and medulla were fixed by immersion in buffered formalin (pH 7.4), dehydrated and embedded in paraffin [43]. Sections of 3 μm were stained with hematoxilin and eosin. The histological profile of twenty proximal tubules randomly selected per rat (6 rats per experimental group) was recorded using a Leica Qwin Image Analyzer (Cambridge, England). The percentage of tubular area with histopathological alterations like swelling, cytoplasmic vacuolization, desquamation or necrosis was obtained. The percentage of damaged area of Cis and Cis+FeTPPS groups was compared.

### Immunohistochemical localization of 3-nitro-L-tyrosine (3-NT)

For immunohistochemistry, 3 μm sections were deparaffined with xylol and rehydrated with ethanol. Endogenous peroxidase was quenched/inhibited with 4.5% H_2_O_2 _in methanol by incubation for 1.5 h at room temperature. Nonspecific adsorption was minimized by leaving the sections in 3% bovine serum albumin in phosphate buffer saline for 30 min. Sections were incubated overnight with a 1:700 dilution of anti-3-NT antibody. After extensive washing with phosphate buffer saline, the sections were incubated with a 1:1000 dilution of a peroxidase conjugated anti-rabbit Ig antibody for 1 h, and finally incubated with hydrogen peroxide-diaminobenzidine for 10 s. Sections were counterstained with hematoxilin and observed under light microscopy. All the sections from the four studied groups were incubated under the same conditions with the same antibodies concentration, and in the same running, so the immunostaining was comparable among the different experimental groups [43].

### Statistics

Results are expressed as the mean ± SEM. Data were analyzed by one-way ANOVA followed by Bonferroni's multiple comparisons. Non-paired t-test was used to compare the quantitative histological damage data using the software Prism 3.02 (GraphPad, San Diego, CA, USA). P ≤ 0.05 was considered statistically significant.

## Authors' contributions

YICH performed animal experimentation, biochemical determinations, statistical analyses, light microscopy and immunohistochemical studies. RHP supported the light microscopy and immunohistochemical studies. JPCH conceived, designed and coordinated the study. All authors read and approved the final manuscript.
